# Calreticulin and other components of endoplasmic reticulum stress in rat and human inflammatory demyelination

**DOI:** 10.1186/2051-5960-1-37

**Published:** 2013-07-15

**Authors:** Mary Ní Fhlathartaigh, Jill McMahon, Richard Reynolds, David Connolly, Eibhlín Higgins, Timothy Counihan, Una FitzGerald

**Affiliations:** 1 National Centre for Biomedical Engineering Science, Galway Neuroscience Centre, National University of Ireland, Galway, Ireland; 2 Centre for Neuroscience, Department of Medicine, Imperial College, London, UK; 3 University Hospital, Galway, Ireland

**Keywords:** Calreticulin, ER stress, EAE, Spinal cord demyelination, Multiple sclerosis

## Abstract

**Background:**

Calreticulin (CRT) is a chaperone protein, which aids correct folding of glycosylated proteins in the endoplasmic reticulum (ER). Under conditions of ER stress, CRT is upregulated and may be displayed on the surface of cells or be secreted. This ‘ecto-CRT’ may activate the innate immune response or it may aid clearance of apoptotic cells. Our and other studies have demonstrated upregulation of ER stress markers CHOP, BiP, ATF4, XBP1 and phosphorylated e-IF2 alpha (p-eIF2 alpha) in biopsy and post-mortem human multiple sclerosis (MS) samples. We extend this work by analysing changes in expression of CRT, BiP, CHOP, XBP1 and p-eIF2 alpha in a rat model of inflammatory demyelination. Demyelination was induced in the spinal cord by intradermal injection of recombinant mouse MOG mixed with incomplete Freund’s adjuvant (IFA) at the base of the tail. Tissue samples were analysed by semi-quantitative scoring of immunohistochemically stained frozen tissue sections. Data generated following sampling of tissue from animals with spinal cord lesions, was compared to that obtained using tissue derived from IFA- or saline-injected controls. CRT present in rat serum and in a cohort of human serum derived from 14 multiple sclerosis patients and 11 healthy controls was measured by ELISA.

**Results:**

Stained tissue scores revealed significantly (p<0.05) increased amounts of CRT, CHOP and p-eIF2 alpha in the lesion, lesion edge and normal-appearing white matter when compared to controls. CHOP and p-eIF2 alpha were also significantly raised in regions of grey matter and the central canal (p<0.05). Immunofluorescent dual-label staining confirmed expression of these markers in astrocytes, microglia or neurons. Dual staining of rat and human spinal cord lesions with Oil Red O and CRT antibody showed co-localisation of CRT with the rim of myelin fragments. ELISA testing of sera from control and EAE rats demonstrated significant down-regulation (p<0.05) of CRT in the serum of EAE animals, compared to saline and IFA controls. This contrasted with significantly increased amounts of CRT detected in the sera of MS patients (p<0.05), compared to controls.

**Conclusion:**

This data highlights the potential importance of CRT and other ER stress proteins in inflammatory demyelination.

## Background

Proteins associated with endoplasmic reticulum (ER) stress have recently been shown in human demyelinating lesions in central nervous system white and grey matter [1-4]. Homeostasis within the ER is of great importance as it is the site of synthesis and folding of approximately one third of all mammalian proteins i.e., those which are targeted to membranes or for secretion. The ER also plays a central role in lipid synthesis and is the major site of calcium storage within the cell. However, a range of pathologies may cause the break-down of protein-folding mechanisms, resulting in a cellular response called the ‘unfolded protein response’ (UPR) or ‘ER stress’ [5].Triggered initially when the ER chaperone GRP78/BiP detaches from ER trans-membrane sensors pancreatic PKR-like ER kinase (PERK), inositol-requiring enzyme 1 (IRE1) and activating transcription factor 6 (ATF6), the UPR temporarily halts protein translation, degrades mis-folded proteins and newly synthesises ER chaperones to restore normal protein folding. Detection of raised levels of Grp78 / BiP, X-box-binding factor 1 (XBP1), phosphorylated PERK (p-PERK), phosphorylated IRE1 (p-Ire1) or phosphorylated eukaryotic initiation factor alpha (p-eIF2α) is indicative of an active ‘protective’ ER stress response. On the other hand, increased expression of transcription factor CHOP is widely considered to indicate that cells may have activated a pro-apoptotic response following the failure to restore normal ER function [6].

Calreticulin (CRT) is a molecular chaperone normally associated with the CRT-calnexin glycoprotein-folding machinery and calcium binding within the ER [7]. However, there is an increasingly diverse array of physiological and pathological functions now associated with cell surface or secreted CRT [8,9]. For example, cell surface CRT has a putative role in the clearance of apoptotic cells [10,11] and in the activation of the innate immune response [12,13]. The fact that it is induced following ER stress and the reported link between CRT and autoimmunity, in the context of rheumatoid arthritis [14], prompted us to examine the expression of CRT and other markers of ER stress in an experimental autoimmune encephalomyelitis (EAE) model of spinal cord demyelination. Pathology was induced in Dark Agouti (DA) rats in response to intradermal injection of recombinant mouse myelin oligodendrocyte glycoprotein (rmMOG) and the expression of CHOP, BiP, p-eIF2α, XBP1 and CRT examined. Levels of circulating CRT in the sera of diseased rats and in MS patient sera were also quantified. The potential relevance of CRT and other ER stress marker protein expression in the development of a demyelinating phenotype is discussed.

## Results

### Significant up-regulation of protein markers of ER stress in demyelinating white matter

**Figure 1:**
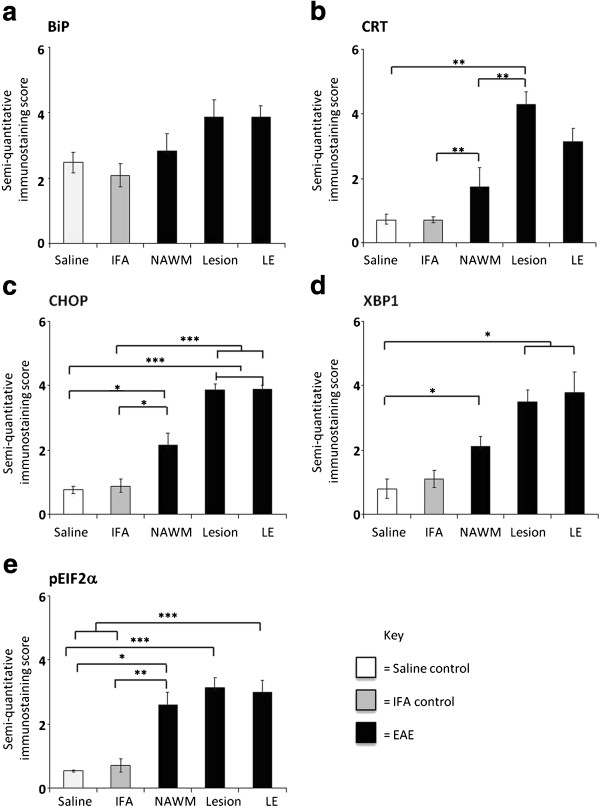
**Quantification of up-regulation of protein markers of ER stress in spinal cord EAE lesions. **Semi-quantitatitive analysis of the spinal cord white matter EAE lesions and lesion edges revealed a trend towards upregulation of BiP, compared to IFA and saline controls **(a)**. Scores for CRT **(b)**, CHOP **(c) **and p-eIF2α **(e) **were significantly higher within lesions and the NAWM than those measured in the NAWM of IFA samples. Significant upregulation of CRT, CHOP, XBP1 **(d) **and p-eIF2α, was found in EAE lesions and NAWM, when compared to saline controls. Key: * = p<0.05; ** = p<0.01; *** = p<0.001. Data is represented as mean arbitrary units (AU) ± SEM.

**Figure 2:**
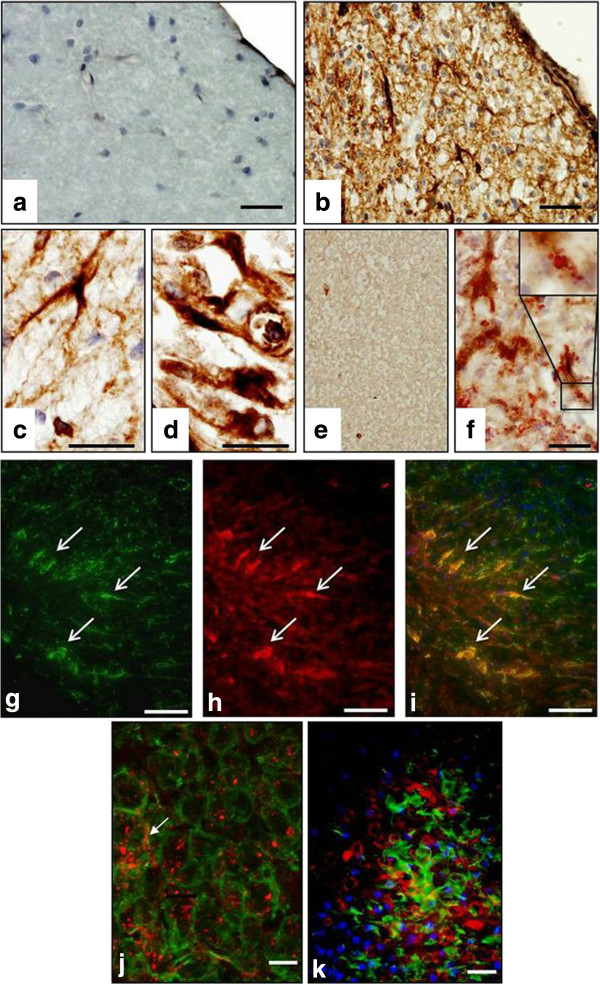
**CRT staining in spinal cord EAE lesions. **There was little or no CRT expressed in IFA-injected spinal cords **(a) **compared to EAE lesions **(b)**. At increased magnification, CRT staining was evident in a number of cell types, morphologically resembling astrocytes **(c) **and macrophages **(d)**. CRT was expressed in Oil Red O deposits **(f **and digitally zoomed inset). ORO deposits colocalising with CRT were absent from IFA control spinal cord tissue **(e)**. Fluorescent dual-labelled images **(g**-**i) **show CRT (red) expression in astrocytes (GFAP, green), as well as macrophage/microglia (Iba1^+^, green, **k)**. Confocal analysis demonstrated punctate staining of CRT in microglia **(j)**. Scale bars = 60 μm **(g**-**i)**, 50 μm **(a**-**b**,**e**-**f**,**j**-**k)**, 20 μm **(c**-**d)**.

Immunohistochemical staining confirmed a significantly higher degree of expression of CRT, CHOP, XBP1 and p-eIF2α within demyelinating lesions, when compared to normal IFA- and saline-injected controls (Figure 1). CRT achieved a semi-quantitative score of 4.3 ± 0.4 arbitrary units (AU), in lesion centres, against scores of 0.7 AU (± 0.1-0.2, p<0.01) in the NAWM of IFA- or saline-injected animals. The CRT score dropped slightly (not significant) to 3 AU at the lesion edge. Sample low-magnification images of CRT-stained control and experimental tissue are shown in Figure 2a and 2b. Morphological criteria applied to single chromogenic labelling suggested that different proportions of a range of cell types were expressing CRT (Figure 2b, c and d). This was confirmed by dual immunofluorescent labelling of astrocytes and microglia (Figure 2g-k). Double-staining of myelin fragments and CRT demonstrated that in some areas of lesioned tissue, CRT was colocalising with the lipid rim (Figure 2f).

In contrast to CRT, monitoring of changes in BiP expression within different tissue regions yielded no significant results (Figure 1).

**Figure 3:**
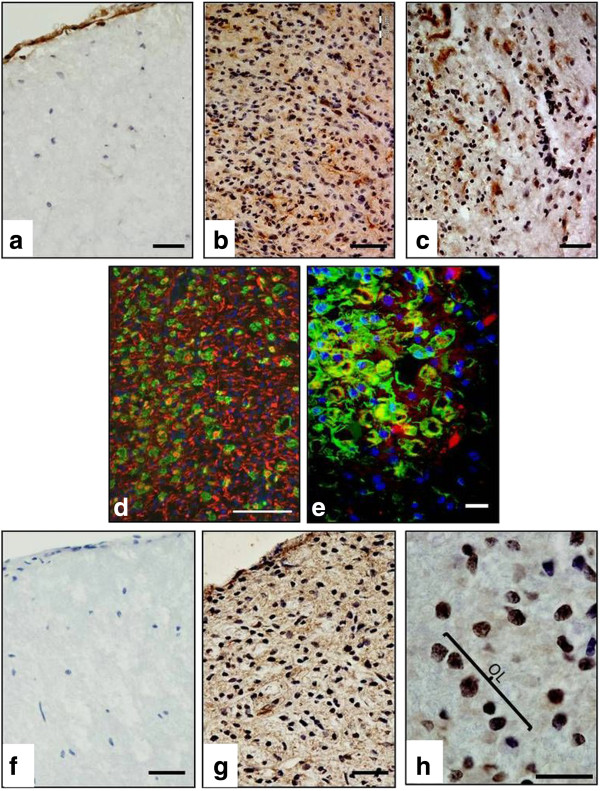
**Immunohistochemical staining of CHOP and XBP1 expression in spinal cord EAE lesions. **CHOP and XBP1 were positively expressed in spinal cord EAE lesions **(b** &**g**, respectively) but were minimal or absent in IFA control tissue **(a **&**f)**. At a higher magnification **(h)**, clear positive/nuclear staining of cells with morphological ‘street-like’ features of oligodendrcoytes is present. A ventro-lateral white matter lesion dual-labelled using antibodies to SMI32, NeuN and CHOP shows a large number of CHOP-positive cells with features consistent with microglia/macrophages within the lesion centre **(d)**. Co-localisation of CHOP in macrophage/microglia was confirmed (see ‘**e**’) by dual immunofluorescence (Iba1^+^/green, CHOP/red). Scale bars = 100 μm **(d)**, 50 μm **(a**-**c**; **e**, **f**-**g)**, 20 μm **(h)**.

When staining for transcription factor CHOP was carried out (see Figure 3a and 3b), it revealed significantly higher (p<0.001) expression in the lesion (3.9 AU ± 0.2) and lesion edge (3.9 ± 0.1), when compared to saline (0.8 ± 0.1) or IFA (0.9 ± 0.2) -injected controls (Figure 1). Tissue stained for XBP1 (see Figure 3g and 3h) had a slightly lower profile of expression, scoring 3.5 ± 0.4 within lesions, rising to 3.8 ± 0.6 at the lesion edge, values which were significantly different (p<0.05) to those calculated for saline controls, but not those obtained when IFA controls were analysed. Representative images of tissue stained with CHOP and XBP1antibodies are provided in Figure 3a -3h. Again, morphological criteria suggest that a variety of cell types express these transcription factors (see Figure 3c, d, h), which, in the case of CHOP, was confirmed by CHOP-IBA1 dual labelling (Figure 3d, e).

**Figure 4:**
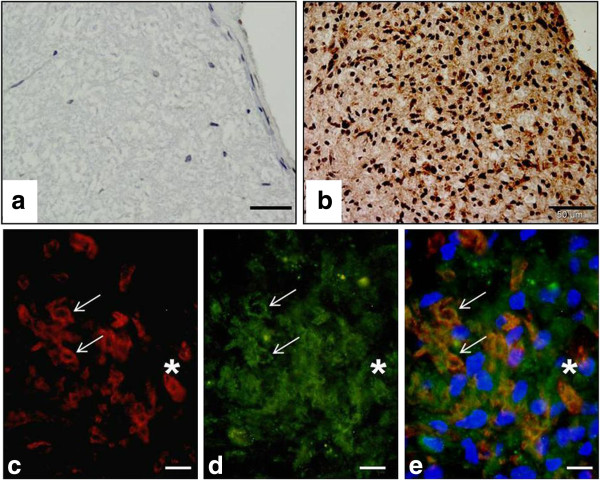
**Profile of expression of p-eIF2αin spinal cord EAE lesions. **p-eIF2α was expressed in spinal cord EAE lesions **(b) **at an increased level compared to IFA control tissue **(a)**. Dual immunofluorescence (asterisk, arrows, CD68 / green, CHOP red) demonstrated that p-eIF2α (green) was expressed in macrophages **(c**-**e)**. Scale bars = 50 μm **(a**-**b)**, 10 μm **(c**-**e)**.

Finally, immunohistochemical analysis of expression of p-eIF2α provided confirmation that the PERK arm of the ER stress response was activated in diseased WM of the spinal cord. Semi-quantitative scores were slightly lower than those seen for the other markers tested, at 3.1 ± 0.3 (lesion) or 3.0 ± 0.4 at the lesion edge. Lesion edge scores differed significantly from those recorded for IFA (0.7 ± 0.2, p<0.05) and saline (0.5 ± 0, p<0.05) controls, while the lesion scores differed significantly from the saline controls only (p<0.05). Staining of a variety of cell types was clearly evident (Figure 4b) and expression in microglia and was confirmed by dual immunofluorescence (Figure 4c-e).

Monitoring of NAWM staining patterns showed that in all cases, protein expression was lower than that seen in the lesion edge and centre. In the case of CRT (1.7 ± 0.5), CHOP (2.2 ± 0.4) and p-eIF2α expression levels differed significantly (p<0.01 or 0.05) from those seen in IFA controls, whereas expression of XBP1 (2.1± 0.3) significantly differed (p<0.05) from that seen in saline controls only.

### Significant up-regulation of protein markers of ER stress in grey matter and central canal of the spinal cord

**Figure 5:**
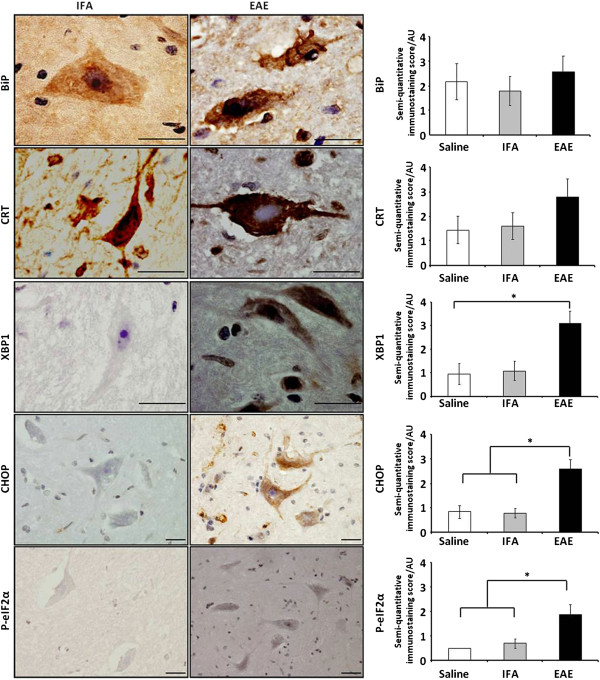
**Upregulation of ER stress molecules in spinal cord grey matter neurons.** Semi-quantitative analysis of ER stress molecule expression in neurons of the grey matter showed an upward trend in BiP and CRT expression, when compared to control IFA or saline groups. CHOP, XBP1 and p-eIF2α scores differed significantly from saline controls, while staining of CHOP and p-eIF2α protein also differed significantly from the pattern seen in IFA controls. Histogram data (RH panels) is represented as semi-quantitative immunostaining score / mean arbitrary units (AU)±SEM, n = 5 for all scores. Key: * = p<0.05. Representative images of staining of the grey matter in IFA controls (LH panels) and EAE samples (middle panels), is shown. Scale bars = 50 μm.

Expression of BiP, CRT, CHOP, XBP1 and p-eIF2α was next examined in the grey matter (GM). Intense staining of motoneurons of the ventral horn by anti-BiP and anti-CRT antibodies was obtained (scoring between 2 and 3), but there was no significant difference in numbers of positively stained cells, when control and experimental tissue was compared (Figure 5). However, significantly increased expression of CHOP (2.6 ± 0.4, p<0.05) was detected, when compared to IFA (0.8 ± 0.2) and saline (0.8 ± 0.3) control specimens. The XBP1 scores (3.1 ± 1.6) differed significantly from those recorded for saline-injected samples (1.1 ± 0.5, p<0.05), differences were not significant when comparisons were made with IFA tissue (Figure 5).

The intensity of chromogenic staining of neurons for p-eIF2α, was consistently higher than that obtained in IFA controls (bottom panels Figure 5) and was lower than that seen for other markers of ER stress. The proportion of cells which stained positively was also lower, a fact reflected in the lower scores of 1.9 ± 0.4 obtained. Nonetheless, these scores were found to differ significantly from levels found in controls which yielded 0.7 ± 0.1 (IFA, p<0.05) or 0.5 ± 0 (saline, p<0.01). Dual immunofluorescent labelling of neurons confirmed co-expression of CHOP (Additional file 1).

**Figure 6:**
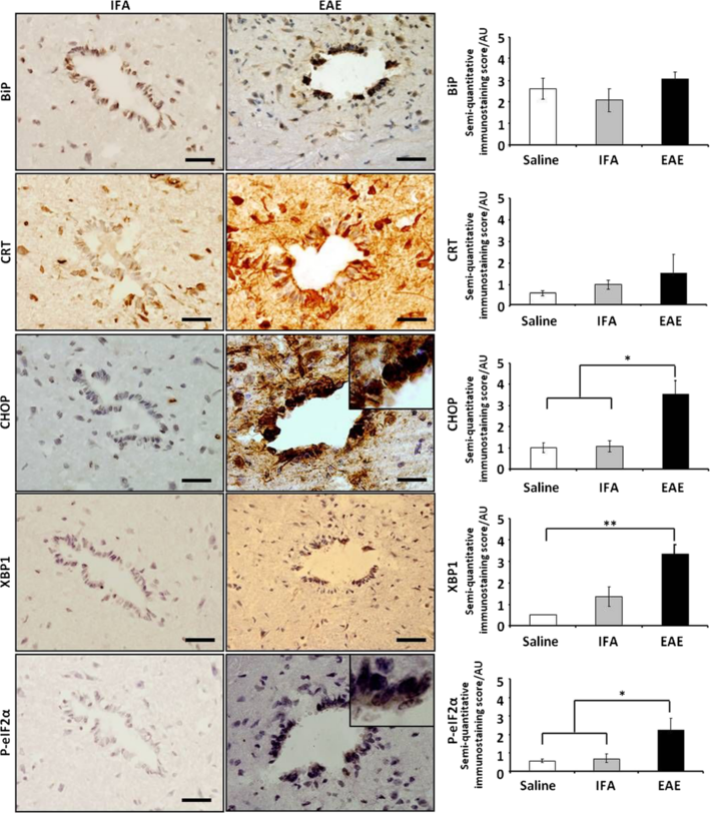
**Upregulation of ER stress molecules in the spinal cord central canal region.** Semiquantitative analysis of the cells of the central canal (RH panel) showed a trend towards upregulation of the ER stress chaperones BiP and CRT when compared to control animals but these differences did not reach significance. Significant upregulation of CHOP, XBP1 and p-eIF2α was detected when EAE and saline samples were compared. CHOP and p-eIF2α values were significantly different to those seen in IFA controls. Representative images of staining in IFA control spinal cord tissue (LH panels) and in EAE lesions (middle panels) are shown. Magnified images of cells positively stained for CHOP (inset, RH middle panel) and p-eIF2a (inset, bottom middle panel) show cuboidal morphology consistent with ependymal cells. Histogram data is represented as semi-quantitative immunostaining score / mean arbitrary units (AU)±SEM, n = 5 for all scores except XBP1, where n = 4 for EAE-treated animals and n = 3 for CRT stained section in saline control group. Key: *=p<0.05; **p<0.01. Scale bars = 50 μm.

Semi-quanitative scoring of staining of the central canal (Figure 6), identified significantly increased expression of CHOP (3.6 ± 0.6, p<0.05), XBP1 (3.4 ± 0.4, p<0.01) and p-eIF2α (2.3 ± 0.6, p<0.05), when compared to the same proteins in the central canal of IFA samples (CHOP, 1.1 ± 0.3; XBP1, 1.4 ± 0.5; p-eIF2α, 0.2 ± 0.1) or tissue from saline controls (CHOP, 1.0 ± 0.2; XBP1, 0.5 ± 0; p-eIF2α, 0.6 ± 0.1).

### Circulating CRT is significantly down-regulated in the serum of EAE animals and is significantly increased in the serum of individuals with MS

**Figure 7:**
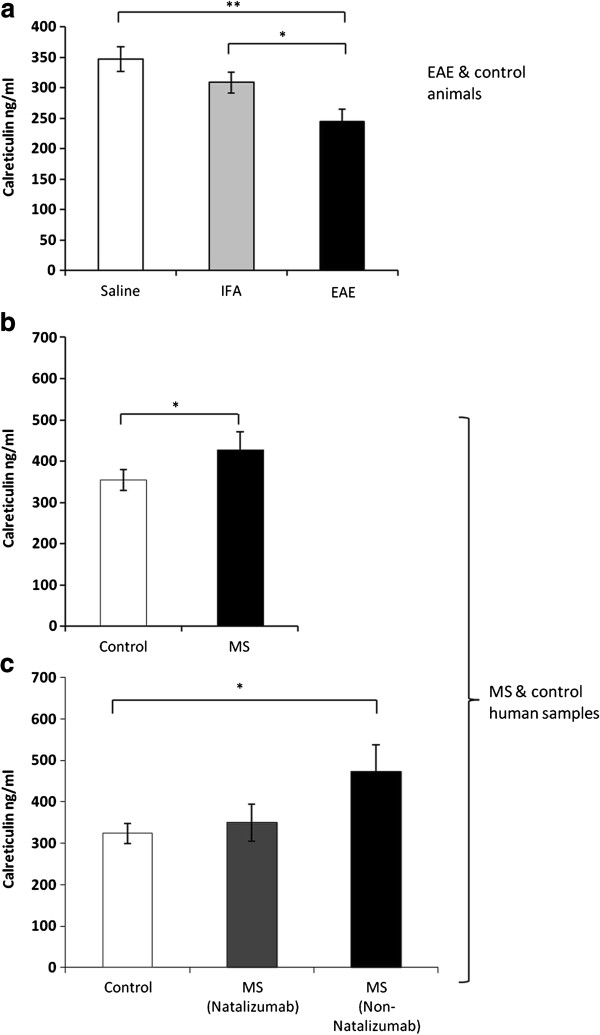
**Quantification of circulating CRT in rat and human sera. **Levels of CRT in rat sera **(a) **were measured by ELISA, revealing that CRT in EAE animals (n=5) was significantly (p<0.05) less than that in control animals (n=5). Testing for CRT in human sera **(b) **revealed significantly increased CRT in human MS sera (p<0.05), n=14) when compared to healthy controls (n=11). When data from MS samples was separated **(c) **according to whether or not individuals were undergoing treatment with nataluzimab, CRT was still significantly higher (p<0.01) in non-nataluzimab-treated patients (n=5), compared to samples from healthy controls (n=11). There was no significant difference in levels found in individuals on nataluzimab when compared to CRT in healthy controls.

Levels of CRT in the serum of EAE animals were analysed by ELISA and compared to values generated by control IFA and saline rats. The 244 ± 21 ng/ml of CRT detected in EAE sera was significantly lower than the 308 – 347 ±18-20 ng/ml found in IFA (p<0.05) or saline (p<0.01) controls (Figure 7a). A significant difference (p<0.05) in the opposite direction was determined when serum samples from 14 individuals suffering from MS were compared to samples taken from 11 non-neurological controls (Figure 7b). That is, a mean level of 428 (± 40) ng/ml was detected in persons with MS (n=14), which was 28% higher than the average 335 (± 17) ng/ml seen in controls (n=11). It is worth noting that none of the MS patients were on steroids at the time of sampling and that in all cases it had been at least 5 months since the last relapse (with the exception of one individual, for whom this information was not available).When data generated by individuals with MS is sub-divided into two groups (Figure 7c), depending on whether or not patients were on Natalizumab, mean CRT levels were lower in Natalizumab-treated patients (382 ± 48 ng/ml, n=9) than in those who were not on Natalizumab (512 ± 67 ng/ml, n=5). Moreover, the mean value for Natalizumab-treated patients was still higher, but no longer differed significantly from healthy control CRT levels, while significant differences (p<0.05) were retained between non-Natalizumab samples and controls (Figure 7c).

## Discussion

The chief outcomes from this study have been the demonstration for the first time that: (i) following MOG-induced spinal cord demyelination in the DA rat, CRT, CHOP and p-EIF2α were present at significantly increased levels within spinal cord lesions; (ii) significantly increased amounts of CRT, CHOP, XBP1 and p-eIF2α were detectable in the region of the central canal of diseased animals; (iii) levels of circulating CRT were significantly lower in EAE rat sera, when compared to control samples; (iv) CRT in human blood sera was present at significantly higher levels in individuals with MS, than in healthy controls.

Using intradermal injection of rmMOG, demyelination was induced in the spinal cord of adult female DA rats. Control animals were injected with IFA or saline only. Nine out of 10 animals developed a phenotype 13–21 days post MOG injection, which was retained in 5 individuals on the day of tissue harvesting. Inflammatory myelin loss was identified by staining of myelin and infiltrating microglia and the presence of myelin fragments within lesions was confirmed using ORO staining. After isolation of total RNA from whole spinal cord lysates, real-time PCR analysis of ER stress-related transcripts did not detect significant differences between control and EAE samples. A semi-quantitative protocol was then applied to immunohistochemically-stained tissue from the 5 rats in which inflammatory pathology was confirmed. On a scale of 1 to 5, the degree of staining was scored within the lesion, lesion edge, NAWM, GM and the region of the central canal and compared to that generated using IFA and saline controls. A total of 240 experimental and control tissue sections were examined, enabling us to profile the expression of BiP, CRT, CHOP, XBP1 and p-eIF2α.

At significantly increased scores, ranging from 1.6 to 5 in all EAE-associated regions examined, CRT was detected in astrocytes, microglia and GM neurons. When present, oligodendrocyte expression of CRT was minimal (not shown). IFA and saline control samples achieved CRT scores of 1 or less. An intriguing finding was the localisation of CRT to the rim of ORO-positive myelin fragments (confirmed in a human MS tissue sample, see additional file 2) and the punctate or ‘patchy’ nature of CRT staining seen when tissue was dual-labelled with CRT and GFAP or IBA1 (Figure 4i and j).

In contrast to CRT, we were unable to detect significant changes in the proportion of BiP-positive cells, when control and EAE samples were analysed. However, with scores of 2.2 to 3.9 AU, compared to staining in saline and IFA sections, which yielded scores of 1 or less, dual-labelling confirmed CHOP expression in astrocytes and microglia. Positive staining for XBP1, which scored 2.1 to 3.5, could be seen in a range of cell types within WM lesions following chromogenic single labelling, but the level of staining differed significantly only from the pattern seen in saline controls.

The final marker of ER stress assessed was p-eIF2α. Following tissue staining, it yielded scores of 1.9 to 3.1 AU. When morphological criteria were applied to the pattern of chromogenic staining seen, p-eIF2α staining appeared to be present in a range of cell types. Dual labelling confirmed p-eIF2α in microglia. Expression of p-eIF2α within lesions, at the lesion edge, in GM and the CC was also significantly higher than levels recorded for equivalent areas in saline and IFA controls.

Results obtained when NAWM was examined hinted that CRT, CHOP and p-eIF2α could be involved in lesion development, as these markers were present at significantly higher levels when compared to control counterparts.

Detection of significantly higher levels of CHOP and phospho-eIF2α within the region of the central canal in diseased animals hints that possible triggers of ER stress may be present in the cerebrospinal fluid and an investigation of candidate molecules is warranted.

We then expanded our investigations to include analysis of CRT secretion into the blood of EAE rats and in samples drawn from a cohort of 14 individuals with MS and 11 healthy controls. Data showed significantly reduced levels of CRT in rat sera when compared to IFA or saline control animals. The reverse was seen when human samples were analysed, in that MS patient samples contained significantly higher levels of secreted CRT. When results generated using samples taken from Natalizumab-treated patients only were analysed separately, mean CRT values were not found to differ significantly from mean values in healthy individuals. In contrast, the amounts of CRT detected in serum from individuals not undergoing treatment with Natalizumab, remained significantly higher than controls.

There are no previous reports of ER stress in rat EAE models. However, in broad terms, the data we have generated in rats confirms and adds to findings published using SJL and C57Bl/6 mice, in that BiP, CHOP, XBP1 and p-eIF2α were reported as significantly altered in these models [15-19] The question of what individual ER stress proteins are doing in the context of demyelination has yet to be answered. Deslauriers et al. [17] showed that EAE occured to the same degree in CHOP^-/-^ mice as in normal controls, suggesting that CHOP is not required to induce inflammatory demyelination in mice. When present, as in our rat model, CHOP may be involved in perpetuating pro-apoptotic signals leading to loss of CHOP-positive cells within lesions. Alternatively, Popko and colleagues have proposed that CHOP is not pro-apoptotic, but is uniquely required to achieve complete remyelination following inflammatory demyelination [20].

The second major transcription factor associated with ER stress is XBP1. We found this highly specific marker of ER stress to be significantly altered in all regions of EAE lesions and in NAWM, when compared to saline-injected controls, but levels of XBP1 in EAE lesions did not differ significantly from those detected in control IFA animals. This is at odds with reported significant transcriptional upregulation of spliced XBP1 in samples isolated from EAE C57/BL mice [17]. This may be due to the fact that the antibody used to screen our tissue does not discriminate between proteins encoded by spliced or unspliced variants, or may reflect the differences between the disease profile in our animal model and theirs. It is most likely that translational arrest is occuring within cells which stained positively for p-eIF2α, as phosphorylation of this molecule is known to interfere with protein translation, as part of a well characterised self-protective mechanism aimed at reducing the accumulation of misfolded proteins within the ER. It would be useful to determine the duration of this molecular event and how timing of its appearance or disappearance within lesions relates to tissue repair or ultimate cell and tissue destruction.

It is likely that, when functioning within the ER, proteins may have a role which differs to that found outside the ER. For example, while in the ER lumen, CRT participates in the CRT-calnexin cycle, ensuring correct folding of glycosylated proteins and trafficking of mis-folded proteins to the ER-associated degredation system. By an as yet undefined means which may occur under conditions of ER stress and may involve removal of the KDEL ER retention sequence [7], CRT could be displayed on the cell surface. This CRT cell surface expression may require interaction with C1q complement and CD91 [21]. On the other hand, given that EAE is a T cell-mediated disease, it is also possible that CRT detected in the tissue samples derived from our EAE rats, was released from granules produced by invading cytotoxic T lymphocytes [22,23]. Whether originating from brain cells, or released from invading T cells, CRT bound to C1q and CD91, may aid recognition and clearance of apoptotic cells by macrophages/microglia [8]. Furthermore, some investigators have demonstrated a direct interaction between phosphatidylserine (PS) and CRT which was maintained when apoptosis caused PS to be ‘flipped’ to the outer leaflet of the plasma membrane [24,25]. It may be that CRT’s appearance at the cell surface precedes PS exposure, as reported for tumour cells [26]. Tarr et al also described ‘punctate clusters’ containing PS and CRT in apoptotic Jurkat T cells, reminiscent of the puctate staining seen in confocal imaging of CRT-stained EAE lesions (Figure 4j). Intriguingly, there is also a puncate quality to some of the CRT staining in the ORO-CRT images reported here. Again, it is possible that PS abnormally displayed on the outer surface of myelin fragments could be bound by CRT as part of a myelin debris clearance process.

Once CRT is secreted into the circulation, it may function in modulating innate and adaptive immune responses. In this regard, a large body of literature is accumulating on the role of CRT in the pathogenesis of rheumatoid arthritis (RA), another autoimmune disorder, Tarr et al [27] detected significantly higher levels of extracellular CRT in the synovial fluid and plasma of rheumatoid arthritis patients. At values between 232 and 623 ng/ml, the quantities of CRT found in the serum of MS patients examined in our study, were 20 to 30 times higher than those reported by Tarr et al, leading to the speculation that circulating CRT could also be playing a role in the pathogenesis of MS. Recent descriptions that various proteins including Hsp70, BiP and αB-crytstallin [28] have “chaperokine” functions suggest that CRT could also be involved in determining the balance between peripheral pro- and anti-inflammatory T cell subsets, although this remains to be definitively established. Our finding that levels of circulating CRT in the serum of EAE animals were significantly lower than levels detected in control IFA and saline animals is challenging to explain. Further time-course experiments need to be carried out in rat and human cohorts, to determine whether or not circulating CRT is, as we propose, a new robust surrogate biomarker of demyelinating disease and a possible ‘chaperokine’ in MS.

## Conclusion

Through detection of significant changes in CRT expression in rat EAE tissue, we have highlighted the potential importance of CRT as a mediator of MS pathogenesis. Dual labelling of myelin fragments and CRT hints that CRT could be involved in myelin clearance. Detection of circulating CRT points to a possible role for this chaperokine in regulating the profile of circulating immune cells. This, together with our novel detection of significantly altered levels of CHOP, CRT and eIF2α in EAE rat spinal cord, highlights a need for the development of models of ER stress which may be manipulated in the field of MS research.

## Methods

### Expression and purification of MOG protein

Recombinant mouse MOG (rmMOG), corresponding to the N-terminal Ig-like extracellular domain of mouse MOG (amino acids 1 -116), was prepared as previously described [29].

### Animals

Female DA rats (Harlan, UK), weighing between 118-161 g, were used for induction of spinal-cord demyelination. Food and water were available *ad libitum* with light:dark cycles 12:12 in a temperature controlled room (20 ± 2°C), with relative humidity of 45–70%. Animals were acclimatised for one week prior to any experimental procedures. All animal work presented in this paper was performed according to the EU Directive 86/609 and the Cruelty to Animals Act 1876 enacted in Ireland by two Statutory Instruments: S.I No 566/ 2002 and S. I. No 613/ 2005. Additionally and in adherence to NUI Galway policies regarding research involving live animals, this work was also authorised/ approved by National University of Ireland Galway Animal Care and Research Ethics Committee (ACREC).

### EAE induction, clinical evaluation and lesion characterisation

Rats (n=10) were anaesthetised via inhalation anaesthesia and injected intra-dermally into the dorsum base of the tail with a total volume of 100 μl containing 25-50 μg rmMOG (diluted in saline) emulsified in incomplete Freund’s adjuvant (IFA; Sigma, Dublin, Ireland) according to previously described protocols [29,30]. Control animals were similarly injected with a total volume of 100 μl IFA (n = 5) or saline (n = 5). In this cohort, this protocol produced a relapsing-remitting disease course in the majority of animals. Animals were weighed and the clinical level of EAE was scored daily. Animals were assigned a clinical score as follows: 0=no abnormality; 0.5= loss of tone in the tip of the tail; 1 =flaccid tail; 2 =single hind-limb paralysis; 3=double hind-limb paralysis; 4= quadriplegia; 5= moribund.

Mean clinical scores obtained in DA rats over a 43-day period following intradermal injection of rmMOG are depicted in Additional file 3a. Of 10 animals injected with 25 – 50 μg of rmMOG, 9 experienced paralysis at some point during the 43-day period and clinical scores ranged from 0.5 to 3. Remission was experienced in some animals, leading to large standard deviations in clinical scores (Additional file 3). Rats injected with IFA or saline alone did not show clinical symptoms, in contrast to those injected with MOG. A humoral response to MOG was also detected in MOG-injected animals (Additional file 3b). A representative image of the disappearance of MOG-specific staining in the white matter of affected animals is shown in Additional file 3c, contrasting with normal MOG levels retained in control saline or IFA groups (Additional file 3d and e). A macrophage-driven pathology was confirmed as present in all lesions or absent in controls, following immunofluorescent staining of large numbers of cells for the macrophage / microglial-specific marker IBA1 (Additional file 3f – h). Involvement of T cells in pathology was also confirmed using antibiodies to CD3 (Additional file 1). Myelin debris-laden areas were also confirmed in demelinating lesions only, by ORO staining (Additional file 3i-k).

Quantitative assessment of ER stress proteins was carried out in 5 rats in which a demyelinating pathology had been confirmed by loss reactivity to antibodies to MOG and by loss of LFB staining. Lateral and ventral lesions incorporating GM regions were present in 4 out of 5 animals, while a prominent dorsal lesion was detected in the fifth animal. It was noted that the pathological features of the dorsal lesion, in terms of infiltrating immune cells, did not differ to features seen in lateral and ventral lesions. A demyelinating pathology could not be detected in the remaining 5 animals. It is possible that pathology was missed, as half of the tissue collected was snap-frozen for use in RNA isolation. For the reasons stated above, these individuals were excluded from further analysis.

### Measurement of humoral reactivity to MOG

To determine titres of anti-MOG antibody in serum, bloods were collected via cardiac puncture following termination and serum analysed by enzyme-linked immunosorbent assay (ELISA). Following clotting at 4°C, serum was separated and collected following centrifugation and stored at -20°C. Briefly, 10 μg / ml rmMOG was added to 96-well plates (Sarstedt, Wexford, Ireland) and incubated overnight at 4°C before blocking with 2% bovine serum albumin (BSA; Sigma, Dublin, Ireland) in PBS for 1 h at room temperature. The sera were diluted (1:400) in 1% BSA / PBS and incubated for 2 h. Rat IgG–specific alkaline phosphatase-linked secondary antibody (1:5000 in 1% BSA/PBS; Sigma, Dublin, Ireland) was incubated for 1 h, prior to detection with p-Nitrophenyl phosphate (Sigma, Dublin, Ireland). The optical density was measured at 405 nm. All incubations were at 37°C unless otherwise stated.

### Tissue harvesting and processing

Animals were sacrificed by CO_2_ asphyxiation 43 days post-inoculation. Spinal cords (average length 80 mm) were cut into 8 equally sized pieces. Each piece was further bisected, with one piece being snap-frozen for RNA analysis and the second used for histological analysis.

### Histological analysis and immunohistochemistry

All tissue for histology was fixed in freshly prepared 4% paraformaldehyde overnight at 4°C and cryoprotected in 30% sucrose in PBS for 48 h. Tissue was then embedded in OCT (VWR International Ltd, Dublin, Ireland) and stored at -80°C. Histological evaluation was performed on 10 μm frozen sections which were thaw-mounted onto Superfrost Plus slides (Fisher Scientific, Dublin, Ireland) and stored at -80°C. Details of antibodies and conjugates used for immunohistochemistry are shown in Table 1.

**Table 1:** Antibodies and conjugates used for immunohistochemistry

**Antibody/Conjugate**	**Isotype/Species**	**Supplier**	**Dilution**
MOG	Mouse mAb	Prof R Reynolds, Imperial College London	1/100
BiP/GRP78	Rabbit pAb	Abcam (ab32618)	1/100
CRT	Mouse mAb	Abcam (ab22683)	1/1000
CHOP/GADD153	Mouse mAb	Cell signalling (L63F7)	1/200
CHOP/GADD153	Rabbit pAb	Santa Cruz biotechnology (sc793)	1/200
p-EIF2a	Rabbit mAb	Cell signalling (119A11)	1/50
XBP1	Rabbit pAb	Santa Cruz biotechnology (sc-7160)	1/100
Iba1	Rabbit pAb	Wako (019-19741)	1/1000
GFAP	Rabbit pAb	Dako (Z0334)	1/1000
GFAP	Mouse mAb	Sigma (G3893)	1/500
Olig2	Mouse mAb	Millipore (MABN50)	1/75
Olig2	Rabbit pAb	Chemicon(AB9610)	1/65
NeuN	Mouse mAb	Millipore (MAB377)	1/1000
CD68	Mouse mAb	Calbiochem (CB1014)	1/65
SMI32	Mouse mAb	Abcam (ab28029)	1/1000
Biotinylated anti-rabbit Ig	Swine pAb	Dako (E0353)	1/400
Biotinylated anti-mouse Ig	Rabbit pAb	Dako (E0354)	1/400
Biotinylated anti-rabbit IgG (H&L)	Goat pAb	Vector (BA-1000)	1/200
Biotinylated anti-mouse IgG (H&L)	Goat pAb	Vector (BA-9200)	1/200
Alexafluor 488 anti-rabbit	Goat pAb	Invitrogen (A11008)	1/1000
Alexafluor 568 anti-rabbit	Goat pAb	Invitrogen (A11011)	1/1000
Alexafluor 488 anti-mouse	Goat pAb	Invitrogen (A11059)	1/1000
Alexafluor 568 anti-mouse	Goat pAb	Invitrogen (A11019)	1/1000
Fluorescein Avidin DCS	n/a	Vector (A-2011)	1/500
Cy3-conjugated streptavidin	n/a	Jackson Immuno Research	1/4000

### Colorimetric immunohistochemistry

All sections were brought to room temperature, air-dried and endogenous peroxidases blocked by incubation in 1% hydrogen peroxidase in methanol. All primary antibodies were incubated on sections at 4°C overnight, detected using biotinylated secondary antibodies (see Table 1) and ABC horseradish peroxidise-labelled Vectastain Elite ABC reagent (Vector). Diaminobenzidine(DAB) (Dako, Cambridgeshire, UK) was used as chromogen, and all sections were counterstained in haematoxylin.

### Oil red O staining

Sections were brought to room temperature, air-dried and incubated for 4 min in 60% ethanol. Slides were incubated in Oil Red O (ORO; Sigma, Ireland) solution (0.5% ORO in 60% ethanol) for 1 h at room temperature and differentiated by rinsing in 60% ethanol and 4 washes in water. After counterstaining in haematoxylin, slides were mounted in gelatine mounting medium (in-house).

### Immunohistochemistry/ORO double stain

Double-staining for CRT and ORO was carried out by firstly performing standard immunohistochemistry (as described above), prior to counterstaining in haematoxylin and staining with ORO (as described above).

### Immunofluorescent double-staining

All sections were brought to room temperature, air-dried, washed in PBS/0.05% Triton-X-100 and incubated in ice-cold methanol for 10 min. All sections were blocked in 5% normal goat serum (NGS) for 1 h and incubated overnight with primary antibody diluted in 1% NGS/1% BSA. Antibodies were visualised using the relevant Alexafluor conjugates (see Table 1) and all slides mounted in Vectashield containing diamino-2-phenylindole (DAPI) (Vector) to allow visualisation of nuclei.

Prior to carrying out confocal analysis of dual-labelled tissue, signal from one of the primary antibodies was amplified. Staining was done as described above, except a biotinylated primary antibody was used, followed by an appropriate secondary antibody (see Table 1 for details).

### Imaging

UV microscopy images were acquired using Olympus BX51 Upright Fluorescent Microscope (Olympus, Dublin, Ireland) with Improvision Optigrid System, or an Olympus IX81 Fluorescent Microscope, together with Improvision Volocity software (PerkinElmer, Dublin, Ireland). Confocal analysis was done using a Zeiss LSM 510 Axiovert Inverted Confocal Microscope (Carl Zeiss).

### Semiquantitative scoring of tissue staining

Expression of BiP, CHOP, XBP1, p-eIF2α and CRT was assessed in the lesion (L) and lesion edge (LE). It was also noted whether there was positive staining of ER stress-associated molecules in the grey matter (GM), the central canal (CC) and normal appearing white matter (NAWM) adjacent to lesioned WM. Scoring of immunostaining was carried out using a graded scale, ranging from 0.5– 4. Scores were assigned as follows: 0.5=minimal positive staining; 1=small numbers of positive cells in the white matter; 2=moderate number of positive cells 3=moderately high numbers of positive cells; 4=high numbers of positive cells.

### Calreticulin ELISA

Serum levels of CRT were determined by ELISA following the manufacturer’s instructions (USCNK, Life Science Inc, Wuhan, China; catalogue number E91486Hu, for human samples; catalogue number CSB-E14943r for rat samples). Rat serum samples analysed for MOG reactivity were also assayed for expression of circulating CRT.

Clinical details of MS patients sampled for analysis of circulating CRT, including age, disease duration, treatment and time since last relapse are provided in Table 2. A summary of relevant information from age-matched controls is given in the same table.

**Table 2:** **Clinical** &**demographic details of MS** &**control cases**

		**MS cases**					**Control cases**	
**Lab I.D.**	**Sex/Age (y)**	**Disease**	**MS type**				**Lab ID**	**Sex/Age (y)**
		**Duration (y)**		**Last relapse**	**Steroids (Y/N)**	**Treatment**		
MS 11/11	M/33	3	RR	5 mo	N	Natalizumab	C2/11	F/45
MS 16/11	F/33	1	RR	1 yr	N	Copaxone	C4/11	F/28
MS 19/11	F/36	3	RR	3 yr	N	Copaxone	C7/11	M/22
MS 24/11	F/32	12	RR	2 yr	N	Naltrexone	C13/11	M/49
MS 31/11	M/49	3	RR	1 yr	N	Natalizumab	C14/11	F/30
MS 32/11	F/44	22	RR	2.5 yr	N	Natalizumab	C16/11	F/33
MS 33/11	F/39	12	RR	1 yr	N	Natalizumab	C17/11	M/31
MS 35/11	F/24	6	RR	2 yr	N	Natalizumab	C23/11	F/82
MS 36/11	F/33	10	RR	2 yr	N	Natalizumab	C24/11	F/39
MS 38/11	F/46	13	RR	3.5 yr	N	Natalizumab	C2/12	F/24
MS 39/11	M/33	6	RR	3.5 yr	N	Natalizumab	C5/12	F/42
MS 41/11	M/29	3	RR	2 yr	N	Natalizumab		
MS 44/11	F/58	22	RR/SP	NK	N	Copaxone		
MS 47/11	F/48	5	RR	5 yr	N	Rebif		

Key: *mo *months, *yr *years, *NK *not known.

### Statistical analysis

Differences in data sets were determined using analysis of variance (ANOVA) or Student’s t-tests. Real time PCR data was analysed by a one-way ANOVA. Differences in ER stress ELISA data sets were calculated using Newman-Keuls multiple comparisons test whereas Dunn’s post-test was used to evaluate differences for the MOG ELISA data set. Differences in ER stress expression in spinal cord tissue following semi-quantitative analysis was completed with a Kruskal-Wallis test followed by a Dunn’s post test, using GraphPad Prism software (San Diego, Ca, USA). All data were expressed as mean and the standard error of the mean (SEM). Differences were deemed significant if p < 0.05.

## Abbreviations

ATF4: Activating transcription factor 4; ATF6: Activating transcription factor 6; BiP: B cell immunoglobulin binding protein; CRT: Calreticulin; CHOP: C/EBP homologous binding protein; EAE: Experimental autoimmune encephalomyelitis; p-eIF2α: Phosphorylated eukaryotic initiation factor 2 alpha; PERK: PKR-like endoplasmic reticulum kinase; IFA: Incomplete Freund’s adjuvant; IRE1: Inositol requiring enzyme 1; rmMOG: Recombinant mouse myelin oligodendrocyte glycoprotein; XBP1: X-box binding protein 1.

## Competing interests

The authors declared that they have no competing interest.

## Authors’ contributions

MNíF carried out all of the animal work, the real-time PCR, the immunocytochemistry and the ELISAs, in cooperation with JMcM; JMcM managed collection of serum samples from healthy controls; RR, trained MNíF and other group members in the setting up of the EAE model; David Connolly did the confocal microscopy, EH collected and processed all patient blood samples for storage; TC facilitated collection of all patients samples at University College Hospital and advised on study design. All authors read and approved the final manuscript.

## Authors’ information

The EAE and ELISA analyses were carried out by MNíF, to fulfull requirements of her PhD. The work described represents the first EAE study carried out by this group and the first characterisation of ER stress signalling in a rat EAE model. JMcM is a senior postdoctoral researcher in the group, who has extensive experience in histological analysis of postmortem human brain tissue, in particular, that derived from patients with multiple sclerosis. RR is a Professor of Cellular Neurobiology, based in Imperial College London. During a sabbatical stay at NUIG in 2009, RR helped the group to establish the spinal cord EAE model of inflammatory demyelination. RR is well known within the EAE and MS fields. DC is an experimental officer with responsibility for training and maintenance of imaging facilities within the NCBES, NUIG. He has extensive experience in a range of microscopical and imaging techniques. EH was a 3^rd^ year medical student in receipt of a Wellcome Trust summer studentship, who helped us to initiate a new biomarker activity within our research group. TC is a consultant neruologist based at University Hospital Galway who looks after multiple sclerosis patients attending neurology clinics. He is part of the recently initiated MS Biomarker activity at NUIG.UF became an independent investigator after getting an academic appointment at NUIG in 2006. She has been senior author on 3 studies of ER stress in post-mortem human MS brain tissue. This study is the first one done by the group using the EAE animal model.

## Supplementary Material

Additional file 1 **CD3 and CRT staining in EAE spinal cord. **Immunofluorescent labelling of T cells **(a) **within a demyelinated lesion, using CD3 antibody (Abcam ab5690) incubated overnight at 4°C. After washing and re-probing with fluorescently-tagged anti-rabbit secondary antibody, positively stained T cells were detected (green). Dual labelling of grey matter in EAE sample spinal cord tissue **(b) **demonstrated localisation of CHOP in neurons staining positively for NeuN (arrow). The same protocol used for CD3 staining was followed for fluorescent CHOP (Santa Cruz, Sc793) and NeuN (Millipore MAB377) staining. Staining obtained in the absence of primary body, but the presence of anti-rabbit **(c) **or anti-mouse **(d) **secondary antibody is shown. Scale bars = 500 μm **(a**, **c**, **d) **or 50 μm **(b)**. Click here for file

Additional file 2 **CRT expression in MS spinal cord tissue. **Snap-frozen human spinal cord tissue was isolated after a 22 h postmortem delay, from a male who suffered from secondary progressive MS and died at the age of 45 following a disease duration of 15 years. Staining with MOG antibody (hybridoma supernatant from Reynolds lab) detected using biotinylated HRP-labelled anti-mouse secondary antibody and DAB chromogenic substrate, revealed a loss of MOG expression, consistent with a lateral lesion (dotted black line, L, a). Following the same protocol to that used for MOG staining, a serial section was stained for CRT using Abcam ab22683 antibody. Positive staining for CRT was found within the lateral lesion (b). At higher magnification, co-localisation of CRT in or at the rim of Oil Red O-stained myelin fragments was seen (**c **and **d**). Scale bars: 500 μm **(a**-**b)**; 50 μm (**c **and **d**). MS tissue was supplied by the UK Multiple Sclerosis Tissue Bank, funded by the Multiple Sclerosis Society of Great Britain and Northern Ireland, registered charity 207495. Click here for file

Additional file 3 **Clinical scores, humoral response and spinal cord demyelination following induction of EAE.** Clinical signs of paralysis were monitored daily **(a) **following EAE induction. Blood samples were taken following sacrifice and a MOG ELSIA conducted to assess the level of MOG antibodies in the serum. Serum from animals immunised with rmMOG had a significantly higher antibody response than control groups **(b)**. Data are expressed as mean ± SEM. Representative spinal cord neuropathology (43 days post immunisation) with demyelination and infiltrating inflammatory cells (by haematoxylin counterstaining) in lesioned areas **(c) **in DA rats immunized with MOG emulsified in IFA. Intact myelin is seen in the spinal cord white matter of saline- and IFA- injected control rats (“control”; score = 0; **d **&**e**). Immunofluorescent double-staining for MOG (red) and microglia/macrophage (Iba1; green) **(f**-**h) **shows that demyelination is accompanied by macrophage/microglial infiltration of the lesioned area **(f)**. Few Iba1^+ ^cells are present in control animals (**g **and **h**).Tissue from saline- and IFA-injected animals **(j**-**k) **showed no positive staining for ORO. However, there was positive lipid staining in EAE lesions **(i) **indicating the presence of foamy macrophages. Scale bars = 100 μm **(c**-**h) **and 50 μm **(i**-**k)**. Click here for file
