# Supplementary material for: Calreticulin and other components of endoplasmic reticulum stress in rat and human inflammatory demyelination
Source: Acta Neuropathol Commun. 2013 Jul 15;1:37. doi: 10.1186/2051-5960-1-37 (PMC3893522; doi:10.1186/2051-5960-1-37)
Supplement: Additional file 1 — CD3 and CRT staining in EAE spinal cord. Immunofluorescent labelling of T cells (a) within a demyelinated lesion, using CD3 antibody (Abcam ab5690) incubated overnight at 4°C. After washing and re-probing with fluorescently-tagged anti-rabbit secondary antibody, positively stained T cells were detected (green). Dual labelling of grey matter in EAE sample spinal cord tissue (b) demonstrated localisation of CHOP in neurons staining positively for NeuN (arrow). The same protocol used for CD3 staining was followed for fluorescent CHOP (Santa Cruz, Sc793) and NeuN (Millipore MAB377) staining. Staining obtained in the absence of primary body, but the presence of anti-rabbit (c) or anti-mouse (d) secondary antibody is shown. Scale bars = 500 μm (a, c, d) or 50 μm (b). [file 2051-5960-1-37-S1.pdf]

Title: Calreticulin and other components of endoplasmic reticulum stress in rat and human inflammatory demyelination

Journal: Acta Neuropathologica Communications

Authors: Mary Ní Fhlathartaigh, Jill McMahon, Richard Reynolds, David Connolly, Eibhín Higgins, Timothy Counihan, Una FitzGerald[1]

1. Corresponding author: Una FitzGerald, National Centre for Biomedical Engineering Science, National University of Ireland, Galway, Ireland

Email: una.fitzgerald@nuigalway.ie

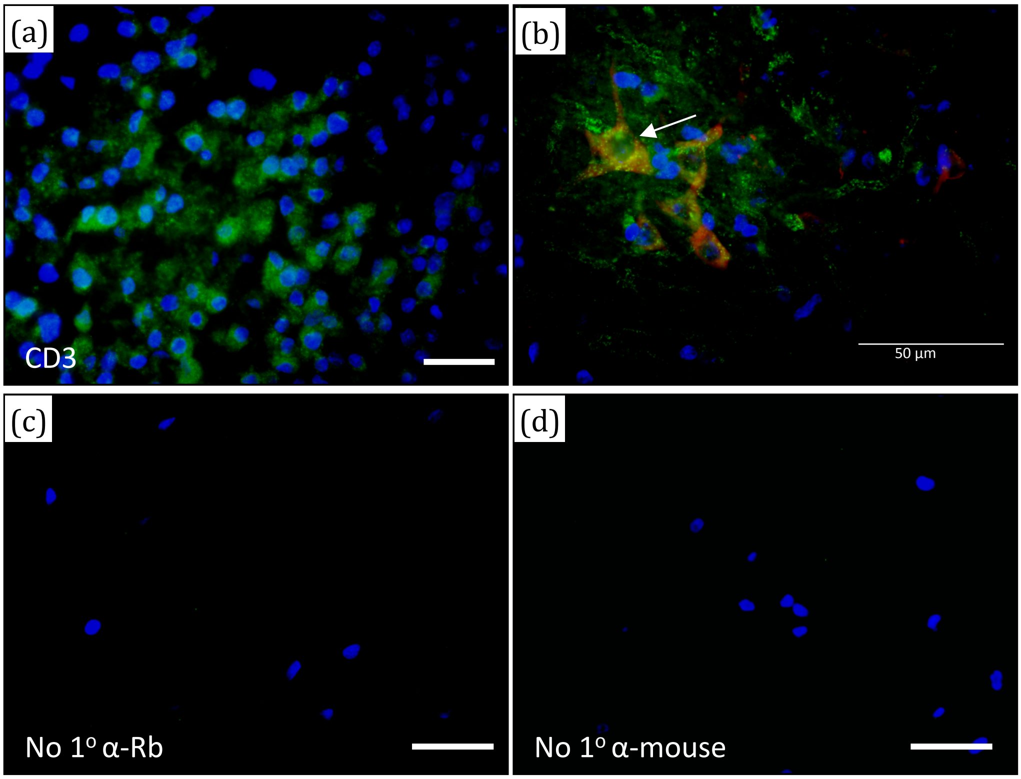

Additional Figure 2: CD3 and CRT staining in EAE spinal cord.
Immunofluorescent labelling of T cells (a) within a demyelinated lesion, using CD3 antibody (Abcam ab5690) incubated overnight at 4°C. After washing and re-probing with fluorescently-tagged anti-rabbit secondary antibody, positively stained T cells were detected (green). Dual labelling of grey matter in EAE sample spinal cord tissue (b) demonstrated localisation of CHOP in neurons staining positively for NeuN (arrow). The same protocol used for CD3 staining was followed for fluorescent CHOP (Santa Cruz, Sc793) and NeuN (Millipore MAB377) staining. Staining obtained in the absence of primary body, but the presence of anti-rabbit (c) or anti-mouse (d) secondary antibody is shown. Scale bars = 500 µm (a, c, d) or 50 µm (b).
