# Supplementary material for: Calreticulin and other components of endoplasmic reticulum stress in rat and human inflammatory demyelination
Source: Acta Neuropathol Commun. 2013 Jul 15;1:37. doi: 10.1186/2051-5960-1-37 (PMC3893522; doi:10.1186/2051-5960-1-37)
Supplement: Additional file 2 — CRT expression in MS spinal cord tissue. Snap-frozen human spinal cord tissue was isolated after a 22 h postmortem delay, from a male who suffered from secondary progressive MS and died at the age of 45 following a disease duration of 15 years. Staining with MOG antibody (hybridoma supernatant from Reynolds lab) detected using biotinylated HRP-labelled anti-mouse secondary antibody and DAB chromogenic substrate, revealed a loss of MOG expression, consistent with a lateral lesion (dotted black line, L, a). Following the same protocol to that used for MOG staining, a serial section was stained for CRT using Abcam ab22683 antibody. Positive staining for CRT was found within the lateral lesion (b). At higher magnification, co-localisation of CRT in or at the rim of Oil Red O-stained myelin fragments was seen (c and d). Scale bars: 500 μm (a-b); 50 μm (c and d). MS tissue was supplied by the UK Multiple Sclerosis Tissue Bank, funded by the Multiple Sclerosis Society of Great Britain and Northern Ireland, registered charity 207495. [file 2051-5960-1-37-S2.pdf]

Title: Calreticulin and other components of endoplasmic reticulum stress in rat and human inflammatory demyelination
Journal: Acta Neuropathologica Communications
Authors: Mary Ní Fhlathartaigh, Jill McMahon, Richard Reynolds, David Connolly, Eibhín Higgins, Timothy Counihan, Una FitzGerald[1]
1. Corresponding author: Una FitzGerald, National Centre for Biomedical Engineering Science, National University of Ireland, Galway, Ireland
Email: una.fitzgerald@nuigalway.ie

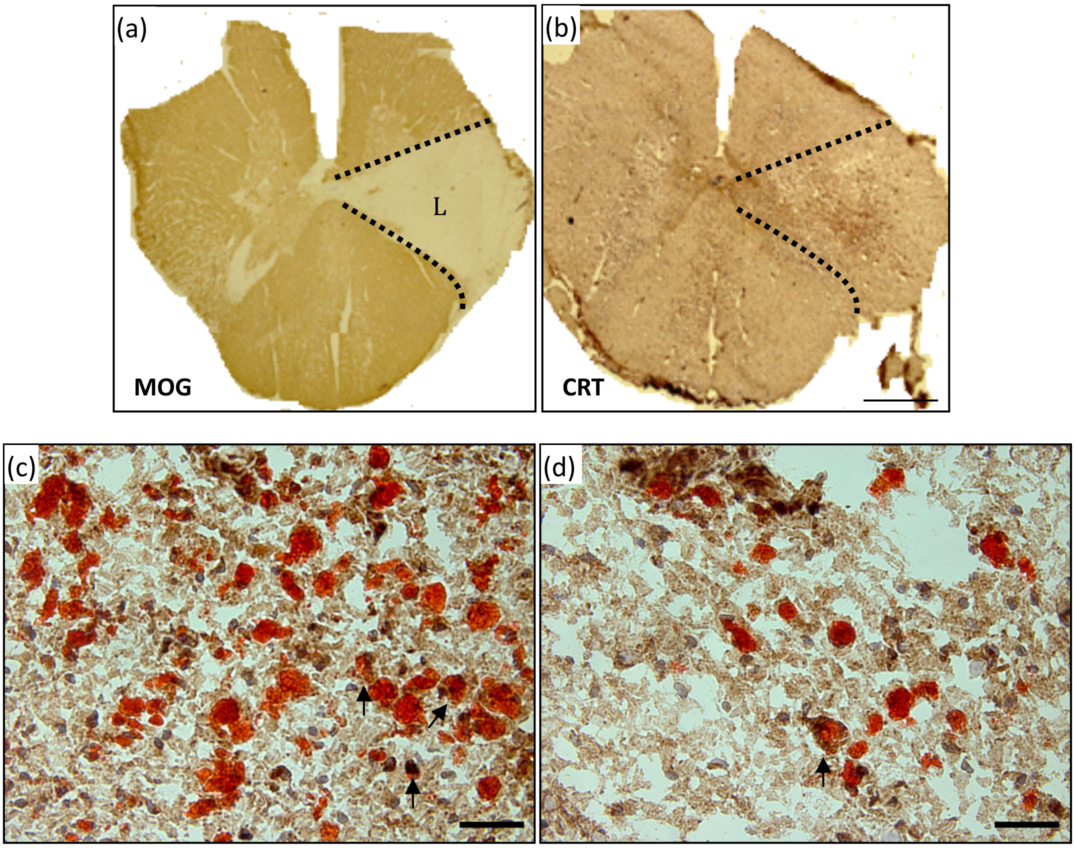

Additional figure 3: CRT expression in MS spinal cord tissue. Snap-frozen human spinal cord tissue was isolated after a 22 h postmortem delay, from a male who suffered from secondary progressive MS and died at the age of 45 following a disease duration of 15 years. Staining with MOG antibody (hybridoma supernatant from Reynolds lab) detected using biotinylated HRP-labelled anti-mouse secondary antibody and DAB chromogenic substrate, revealed a loss of MOG expression, consistent with a lateral lesion (dotted black line, L, a). Following the same protocol to that used for MOG staining, a serial section was stained for CRT using Abcam ab22683 antibody. Postitive staining for CRT was found within the lateral lesion (b). At higher magnification, co-localisation of CRT in or at the rim of Oil Red O-stained myelin fragments was seen (c and d). Scale bars: 500 µm (a-b); 50 µm (c and d). MS tissue was supplied by the UK Multiple Sclerosis Tissue Bank, funded by the Multiple Sclerosis Society of Great Britain and Northern Ireland, registered charity 207495.
