# Supplementary material for: Calreticulin and other components of endoplasmic reticulum stress in rat and human inflammatory demyelination
Source: Acta Neuropathol Commun. 2013 Jul 15;1:37. doi: 10.1186/2051-5960-1-37 (PMC3893522; doi:10.1186/2051-5960-1-37)
Supplement: Additional file 3 — Clinical scores, humoral response and spinal cord demyelination following induction of EAE. Clinical signs of paralysis were monitored daily (a) following EAE induction. Blood samples were taken following sacrifice and a MOG ELSIA conducted to assess the level of MOG antibodies in the serum. Serum from animals immunised with rmMOG had a significantly higher antibody response than control groups (b). Data are expressed as mean ± SEM. Representative spinal cord neuropathology (43 days post immunisation) with demyelination and infiltrating inflammatory cells (by haematoxylin counterstaining) in lesioned areas (c) in DA rats immunized with MOG emulsified in IFA. Intact myelin is seen in the spinal cord white matter of saline- and IFA- injected control rats (“control”; score = 0; d &e). Immunofluorescent double-staining for MOG (red) and microglia/macrophage (Iba1; green) (f-h) shows that demyelination is accompanied by macrophage/microglial infiltration of the lesioned area (f). Few Iba1+ cells are present in control animals (g and h).Tissue from saline- and IFA-injected animals (j-k) showed no positive staining for ORO. However, there was positive lipid staining in EAE lesions (i) indicating the presence of foamy macrophages. Scale bars = 100 μm (c-h) and 50 μm (i-k). [file 2051-5960-1-37-S3.pdf]

Title: Calreticulin and other components of endoplasmic reticulum stress in rat and human inflammatory demyelination.
Journal: Acta Neuropathologica Communications
Authors: Mary Ní Fhlathartaigh, Jill McMahon, Richard Reynolds, David Connolly,Eibhín Higgins, Timothy Counihan, Una FitzGerald[1]
[1.]Corresponding author: Una FitzGerald, National Centre for Biomedical Engineering Science, National University of Ireland, Galway, Ireland .Email: una.fitzgerald@nuigalway.ie

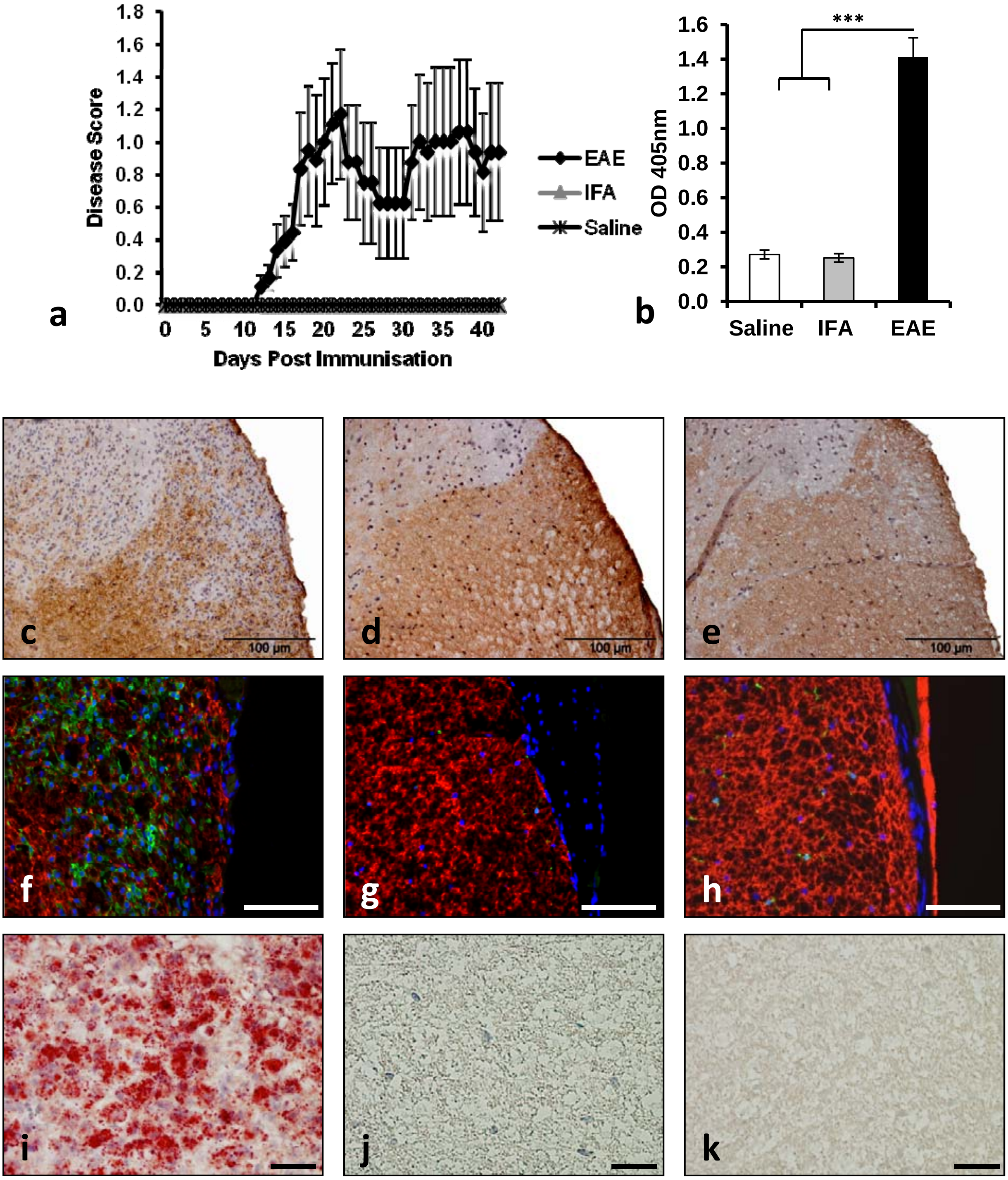

Additonal Figure 1: Clinical scores, humoral response and spinal cord demyelination following induction of EAE. Clinical signs of paralysis were monitored daily (a) following EAE induction. Blood samples were taken following sacrifice and a MOG ELSIA conducted to assess the level of MOG antibodies in the serum. Serum from animals immunised with rmMOG had a significantly higher antibody response than control groups (b). Data are expressed as mean ± SEM. Representative spinal cord neuropathology (43 days post immunisation) with demyelination and infiltrating inflammatory cells (by haematoxylin counterstaining) in lesioned areas (c) in DA rats immunized with MOG emulsified in IFA. Intact myelin is seen in the spinal cord white matter of saline- and IFA- injected control rats ("control"; score = 0; d & e). Immunofluorescent double-staining for MOG (red) and microglia/macrophage (Iba1; green) (f-h) shows that demyelination is accompanied by macrophage/microglial infiltration of the lesioned area (f). Few Iba1^{+} cells are present in control animals (g and h). Tissue from saline- and IFA-injected animals (j-k) showed no positive staining for ORO. However, there was positive lipid staining in EAE lesions indicating the presence of foamy macrophages. Scale bars = 100 µm (c-h) and 50 µm (i-k) .
